# Dosimetric and QA aspects of Konrad inverse planning system for commissioning intensity-modulated radiation therapy

**DOI:** 10.4103/0971-6203.33240

**Published:** 2007

**Authors:** Shrikant Deshpande, V. K. Sathiyanarayanan, Janhavi Bhangle, Kumara Swamy, Sumit Basu

**Affiliations:** Department of Radiation Oncology, Ruby Hall Clinic, Pune, India

**Keywords:** Dose volume histogram, intensity-modulated radiotherapy, multileaf collimator, treatment planning system

## Abstract

The intensity-modulated radiation therapy (IMRT) planning is performed using the Konrad inverse treatment planning system and the delivery of the treatment by using Siemens Oncor Impression Plus linear accelerator (step and shoot), which has been commissioned recently. The basic beam data required for commissioning the system were generate. The quality assurance of relative and absolute dose distribution was carried out before clinical implementation. The salient features of Konrad planning system, like dependence of grid size on dose volume histogram (DVH), number of intensity levels and step size in sequencer, are studied quantitatively and qualitatively.

To verify whether the planned dose [from treatment planning system (TPS)] and delivered dose are the same, the absolute dose at a point is determined using CC01 ion chamber and the axial plane dose distribution is carried out using Kodak EDR2 in conjunction with OmniPro IMRT Phantom and OmniPro IMRT software from Scanditronix Wellhofer. To obtain the optimum combination in leaf sequencer module, parameters like number of intensity levels, step size are analyzed. The difference between pixel values of optimum fluence profile and the fluence profile obtained for various combinations of number of intensity levels and step size is compared and plotted. The calculations of the volume of any RT structure in the dose volume histogram are compared using grid sizes 3 mm and 4 mm. The measured and planned dose at a point showed good agreement (<3%) except for a few cases wherein the chamber was placed in a relatively high dose gradient region. The axial plane dose distribution using film dosimetry shows excellent agreement (correlation coefficient >0.97) in all the cases. In the leaf sequencer module, the combination of number of intensity level 7 with step size of 3 is the optimal solution for obtaining deliverable segments. The RT structure volume calculation is found to be more accurate with grid size of 3 mm for clinical use.

Thus a study regarding various aspects of commissioning of the Konrad inverse planning system for IMRT has been presented, which has been implemented in our clinic.

The present study demonstrates the various aspects of Konrad inverse planning system for its successful commissioning and clinical use. The commissioning of an inverse planning system for step-and-shoot intensity-modulated radiation therapy (IMRT) has many steps - which start from understanding the method by which the algorithm is calculated, the next main steps being collection of data for commissioning the system.[1–4] The department is equipped with the Blue Phantom of Scanditronix Wellhofer with 0.01 cc and 0.13 cc ion chambers for beam data collection.

Once the data has been configured, it is necessary to analyze some of the salient features through which the optimization process proceeds, which results in an objective treatment plan. The dose volume histogram is the most commonly used tool for plan evaluation; therefore, the radiotherapy (RT) structure volume calculation is crucial. The dependence of grid size for RT structure volume calculation was compared for grid sizes 3 mm and 4 mm.[4] The testing of leaf sequencer module[5] is done to arrive at the optimum combination of number of intensity levels and step size. The role of step sizes in optimization is also an important parameter in smoothening the peaks. Further, after putting to use such system, the initial performance of the system is reflected in some of the patient-specific QA regarding the accuracy in commissioning, planning and delivery.

The preliminary report of this study was presented at AMPICON-2006.[1]

## Materials and Methods

Konrad uses the ‘weighted quadratic difference of prescribed and calculated dose distribution’ method[6,7] for the inverse planning algorithm. During optimization, the difference of dose at each voxel in the calculation grid with respect to the objective function is kept at the minimum; and using the constraints and penalty scores, the optimal intensity fluence is calculated. The optimization generates the optimal intensity fluence for each beam direction. The optimized intensity-modulated fields are divided into beamlets that can be delivered by means of sequence of leaf setting by the multileaf collimator (MLC) by leaf sequencer, which is a post-optimization process. For pretreatment verification, various treatment parameters such as beam energy, number of monitor units and multileaf collimator setting have to be verified to ensure the correct dose delivery to the patient. To ensure that what is delivered is same as calculated, the entire plan has been overlaid on the axial image set of the IMRT verification phantom. The entire treatment is delivered to the phantom, and the integrated dose at predetermined points using the CC01 ion chamber is measured in the IMRT phantom (Scanditronix Wellhofer). The measured absolute dose and predicted doses from treatment planning system (TPS) were compared.[8]

‘The film dosimetric QA of the IMRT is carried out by positioning the ready pack EDR2 film at a particular axial image plane and delivering the entire treatment. The axial slice is in the z-axis in the Konrad and at a particular z-level, the film is placed. The dose profile in the DICOM format in that particular z-level, where the film was positioned, is exported to the OminiPro IMRT software. The measured fluence (or planer distribution) and the calculated fluence are compared using the OmniPro IMRT software. The statistical analysis of dosimetric data obtained from film and absolute dosimetry for 37 patients is given in Table 1.

**Table 1:** Statistical analysis of dosimetric data obtained from film and absolute dosimetry for 37 patients

*Statistical parameter*	*Correlation coefficient (Film dosimetry)*	*% absolute dose variation (CC01 ion chamber)*
Mean value	0.9841	2.763
Median	0.9840	2.090
Standard deviation	0.293	2.049
Minimum	0.9734	0.24
Maximum	0.9944	8.0
N	37	37

In the Inverse planning system, the time sequences of MLC settings are derived from an optimal fluence map as a post-optimization process using a software module called ‘Leaf Sequencer.’[5,7] In the leaf sequencer module of Konrad, the planner has two choices. The first one is step size and the other is the number of intensity levels. The effect of these parameters on the optimal and deliverable fluence is studied.

### Choice of intensity levels

The ‘number of intensity levels’ means intensity levels are equally incremented and delivered over a regularly spaced grid. For a higher number of levels, the deliverable solution will be closer to the optimal fluence. However, higher the number of levels, higher will be the number of deliverable segments and low monitor units (MU) segments.

### Choice of step size

The intensity maps of each of the intensity levels are defined over a step size. The typical value is 3. The step size decides the level of smoothening of the fluence profile (i.e., smoothening of isolated high-intensity peaks or deep-intensity valleys), which decreases the complexity of fluence profile.

### Dependence of fluence profile on number of intensity levels

**Figure 1:**
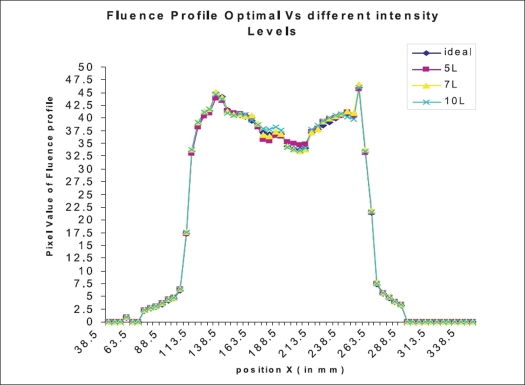
Fluence profile for optimal and with three different intensity levels

In order to study the dependence on number of intensity levels, the ASCII file of the fluence profile of optimal fluence and the fluence obtained with intensity levels 5, 7 and 10 along longitudinal and lateral direction for the same step size are compared. The absolute difference between pixel values (i.e., the dose at the point in that position) at different positions along longitudinal and lateral direction is calculated and plotted [Figure 1]. Also, the numbers of deliverable segments for different intensity values are compared [Table 2].

**Table 2:** Effect of number of intensity levels on number of deliverable segments and low monitor units segments

*No. of intensity levels used*	*No. of segments*	*Maximum absolute error*
5	64	1.469
7	86	1.029
10	118	1.008

**Figure 2:**
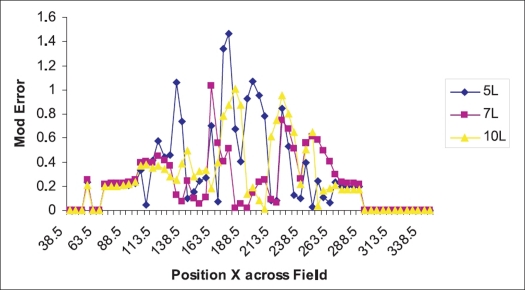
Variation of the mod error across the field width for different intensity levels

The mod value of the difference between the optimal fluence at any given point and values obtained with different intensity levels is given in Figure 2. It is obvious the mod error is higher in the lower intensity levels. The peak of the mod error falls closely as shown in Figure 2.

**Figure 3:**
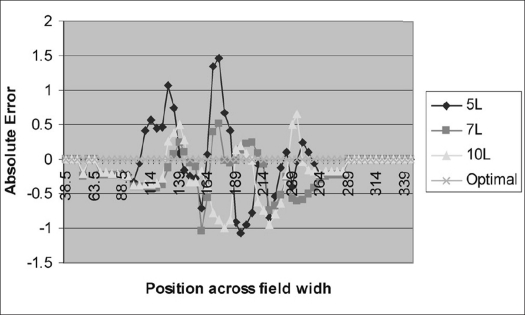
Variation of the mod error across the field width for different intensity levels

Figure 3 depicts absolute error value of the difference between optimal fluence at any given point and values obtained with different intensity levels. The peaks of the error for number of intensity levels 5 and 7 fall in the positive direction, while the peak for number of intensity levels 10 falls in the opposite direction. The frequency distribution of the mod error is given in Table 3.

**Table 3:** Frequency of mod error for different intensity levels

*Intensity level*	*Frequency of mod error (0-0.25)*	*Frequency of mod error (0.25-0.5)*	*Frequency of mod error (0.5-0.75)*	*Frequency of mod error (0.75-1.0)*	*Frequency of absolute error (>1)*
5	41	7	5	6	4
7	42	12	8	0	0
10	38	5	5	5	1

### Dependence of step size on the fluence profile

**Figure 4:**
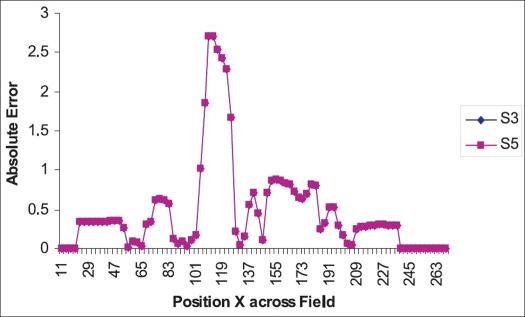
Dependence of step size on fluence profile

Similarly, the step size dependence is studied by keeping the intensity level same, viz., 7, and the ASCII file fluence profile for different step sizes (viz., 3 and 5) and the optimal fluence profile were compared and plotted [Figure 4]. The number of deliverable segments is obtained for different step sizes [Table 5].

**Table 5:** Effect of the step size on number of segments

*No. Step size*	*No. of segments*	*Maximum absolute error*
3	86	2.706
5	86	2.706

Finally, the variation in the absolute volume of an anatomical structure (RT structure) used in the dose volume histogram[2,3,8] analysis, with the change in the reconstruction grid size, is taken up for evaluation.

#### a) Calculation of a precisely known distribution

A simultaneous infield boost plan was created for three concentric doughnut annular structures with known dose to 95% volume of three droughnut annular structures.The cumulative dose volume histogram (DVH) graph shows excellent correlation between dose and volume.

#### b) Variation of structure volume with grid size

The volume of same doughnut annular structures is calculated from CMS-Xio treatment planning system and Konrad inverse planning system for two different grid sizes, viz., 3 mm and 4 mm, and compared. The 3 mm reconstruction grid size shows good agreement between CMS-Xio-calculated and Konrad-calculated volume.

## Results and Discussion

For 37 patients, the absolute dose at a point measured with CC01 chamber varies with respect to calculated value from 0.24% (minimum) to 8.0% the absolute dose variation exceeded 3% in 12 out of the 37 patients [Table 1]; while for 5 out of those 12 patients, the absolute dose variation was within 5%. The correlation coefficient (in case of film dosimetry) was in the range 0.9734-0.9944 for all the patients. The gamma value for all the patients was less than 1.5%. In case of 12 patients wherein variation exceeded 3%, a close study of the isodose distribution revealed that the location of the ionization chambers was in the high dose gradient region. This could be the reason for its variation. In those cases, the measurement point was shifted to the low dose gradient region, and at this point the deviation was found below 3%.

The Oncor Impression Plus accelerator is equipped with Optifocus 82-leaf MLC. Although maximum field size that can be covered is 40 × 40, the maximum size of the target organs that can be covered by the intensity modulated fields is 20 × 20 cm. This is due to the over-travel constraint of the jaws and MLC banks. However, in some situations, the collimator can be rotated to 90°, and this helps in planning for PTV volume wherein the length is around 20 cm.

In the leaf sequencer module, the planner has choices like the number of intensity levels and step size. Planner has to choose the number of intensity levels which finds deliverable solution closer to optimal continuous distribution (Obtained after optimization) and is dosimetrically acceptable. The mod and absolute error between the pixel value of the optimal fluence profile and different intensity levels over the position in x direction (lateral) is calculated and plotted [Figures 2 and 3]. The number of intensity levels ‘7’ gives close matching of deliverable fluence profile to the optimal fluence profile with moderate number of deliverable segments [Tables 2–4]. The MU per segment is always greater than 3 as it fits very well in low MU linearity for Oncor Linear Accelerator, which is dosimetrically acceptable. The step size variation with a given intensity level shows a very minimal effect on the deliverable fluence profile [Table 5 and Figure 4]. Thus we arrived at the conclusion that the combination of number of intensity level ‘7’ with step size ‘3’ forms an optimal solution in leaf sequencer module, which has been incorporated in most of the IMRT plans.

**Table 4:** Statistical analysis of dependence of intensity level on deliverable fluence to optimal fluence

*Statistical parameter*	*Difference between optimal and intensity level 5*	*Difference between optimal and intensity level 7*	*Difference between optimal and intensity level 10*
N	62	62	62
Mean	0.295	0.228	0.266
Minimum	0.00	0	0
Maximum	1.469	1.029	1.008
Range	1.469	1.029	1.008
Median	0.197	0.223	0.198

In DVH plot, the 3 mm grid size shows closer match of structure volume calculation to that of 4 mm reconstruction [Table 6].

**Table 6:** Comparison of the volume of the RT structure with different grid sizes

*Manually calculated volume (in cc)*	*CMS calculated volume (in cc) grid size in mm*		*KonRad reconstruction (in cc) grid size in mm*	
		
	3	4	3	4
64.18	63.84	63.84	65.77	64.32
165.27	165.81	165.81	164.43	148.87
221.13	225.64	225.64	222.83	210.24

## Conclusion

A study regarding various aspects of commissioning of the Konrad inverse planning system for IMRT has been presented. The absolute dose at a point and analysis of the axial dose distribution show good agreement with the accepted criteria.

In the leaf sequencer module, combination of the number of intensity level ‘7’ and the step size ‘3’ is the optimal solution for obtaining deliverable segments. In the dose volume histogram, RT structure volume calculation with grid size 3 mm is found to be accurate for clinical use.
